# Sterol auto-oxidation adversely affects human motor neuron viability and is a neuropathological feature of amyotrophic lateral sclerosis

**DOI:** 10.1038/s41598-020-80378-y

**Published:** 2021-01-12

**Authors:** James C. Dodge, Jinlong Yu, S. Pablo Sardi, Lamya S. Shihabuddin

**Affiliations:** Rare and Neurological Diseases Therapeutic Area, Sanofi R+D, 49 New York Avenue, Framingham, MA 01701 USA

**Keywords:** Neuroscience, Pathogenesis

## Abstract

Aberrant cholesterol homeostasis is implicated in the pathogenesis of amyotrophic lateral sclerosis (ALS), a fatal neuromuscular disease that is due to motor neuron (MN) death. Cellular toxicity from excess cholesterol is averted when it is enzymatically oxidized to oxysterols and bile acids (BAs) to promote its removal. In contrast, the auto oxidation of excess cholesterol is often detrimental to cellular survival. Although oxidized metabolites of cholesterol are altered in the blood and CSF of ALS patients, it is unknown if increased cholesterol oxidation occurs in the SC during ALS, and if exposure to oxidized cholesterol metabolites affects human MN viability. Here, we show that in the SOD1^G93A^ mouse model of ALS that several oxysterols, BAs and auto oxidized sterols are increased in the lumbar SC, plasma, and feces during disease. Similar changes in cholesterol oxidation were found in the cervical SC of sporadic ALS patients. Notably, auto-oxidized sterols, but not oxysterols and BAs, were toxic to iPSC derived human MNs. Thus, increased cholesterol oxidation is a manifestation of ALS and non-regulated sterol oxidation likely contributes to MN death. Developing therapeutic approaches to restore cholesterol homeostasis in the SC may lead to a treatment for ALS.

## Introduction

Amyotrophic lateral sclerosis (ALS) is a progressive neurodegenerative disorder characterized by the selective loss of motor neurons (MNs) in the central nervous system (CNS). Lipid dysregulation is emerging as an important modulator of disease pathogenesis in ALS. For example, several genes implicated in ALS (e.g., ANG, C9ORF72, HNRNPA1, FUS, SIGMAR1, SOD1, TARDBP, VAPB, and VCP) and modifiers of disease progression (e.g., ATXN2 and LXR) are regulators of lipid homeostasis^1–12^. Cholesterol, in particular, may play a pivotal role in disease. Dyslipidemia and altered apolipoprotein metabolism in blood are risk factors for ALS and are also prognostic indicators of disease progression in ALS patients^13–16^. Furthermore, cholesterol esters (CEs) accumulate several fold in the spinal cords of ALS patients^17,18^ and in rodent models of ALS^17–19^, and total cholesterol levels are elevated in the CSF of ALS patients^20^. Importantly, we showed that inducing aberrant cholesterol homeostasis (i.e., cholesterol cacostasis) in the spinal cord of WT mice leads to a robust ALS phenotype featuring CE accumulation, muscular atrophy, astrogliosis, MN death, and reduced survival^18^.

Presumably, increased CE accumulation during ALS manifests as a compensatory response to avoid intracellular toxicity triggered by excess free cholesterol (FC). The neutralization of FC to CE, however, is not completely inert, because CE synthesis leads to a concomitant increase in lysophosphatidylcholine, a lipid that is toxic to human MNs^18^. Thus, additional compensatory responses are likely activated during disease to avert toxicity induced by excess FC. Indeed, we recently reported that enzymatic and transcriptional regulators of cholesterol synthesis are reduced in the spinal cords of ALS patients and SOD1^G93A^ mutant mice^18^. Excess FC removal is also facilitated when FC is hydroxylated to oxysterols and bile acids (BAs) to increase its solubility^21^. Several lines of evidence suggest that FC oxidation may play a role in ALS. For example, oxysterols levels are altered in the blood and CSF of ALS patients^22,23^, and gene variants in liver X receptors (LXRs), transcription factors that lower cholesterol levels upon oxysterol binding, affect disease progression in ALS patients^5^. Moreover, BA precursors affect mouse motor neuron viability through LXR binding^24^ and intermediates of the acidic BA synthesis pathway are reduced in the CSF of ALS patients^20^. Excess FC may also lead to unregulated oxidation when it is inadvertently auto-oxidized following exposure to reactive oxygen species or immune cell oxidizers^25^. Although FC oxidation is implicated in ALS, it is unknown if enzymatically derived or auto-oxidized cholesterol metabolites are increased in the spinal cord during ALS, and if they modulate human MN viability.

Here, we demonstrate that oxidized metabolites of cholesterol are increased in the spinal cords of SOD1^G93A^ mice, a model of familial ALS, and in sporadic ALS patients. Some of these oxidized metabolites of cholesterol were detected in the plasma and feces of SOD1^G93A^ mice as potential biomarkers of ongoing spinal cord cholesterol cacostasis. Notably, human motor neuron viability was adversely affected by auto-oxidized cholesterol metabolites, but not by enzymatically derived oxysterols or BAs. These findings suggest that manifestation of cholesterol cacostasis that occurs during ALS results in increased FC oxidation, and that un-regulated FC contributes to MN death. Thus, restoring CNS cholesterol metabolism to its optimal homeostatic state (i.e., eustasis) may lead to a disease-modifying treatment for ALS.

## Results

### Oxidized cholesterol accumulation occurs in the spinal cord of SOD1^G93A^ mice

**Figure 1:**
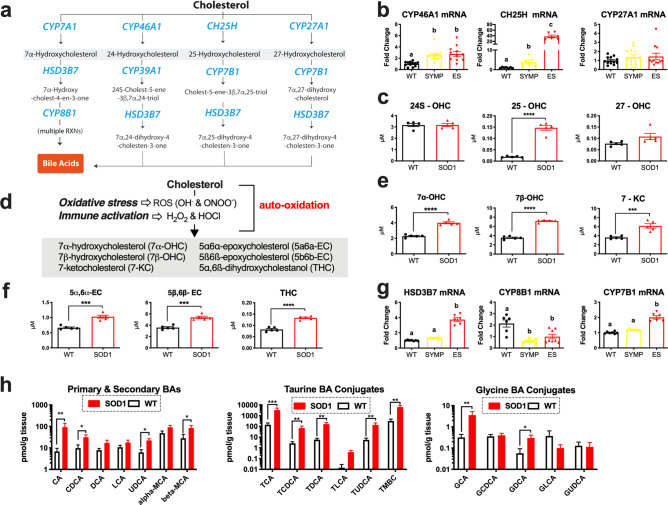
Oxidized metabolites of cholesterol that are elevated in the spinal cords of SOD1^G93A^ mice (**a**) Metabolic pathways summarizing enzymatically derived oxysterol and bile acid synthesis. Disease-related changes in (**b**) CYP46A1, CH25H and CYP27A1 mRNA levels in the lumbar spinal cord of symptomatic (SYMP) and end stage (ES) SOD1^G93A^ mice. (**c**) 25-OHC levels are significantly increased in the lumbar spinal cord of ES SOD1^G93A^ mice. (**d**) Metabolic pathways summarizing the generation of auto-oxidized sterols. (**e,f**) Oxysterols derived from the auto-oxidation of cholesterol are significantly increased in the lumbar spinal cord of ES SOD1^G93A^ mice. (**g**) Enzymes that regulate bile acid synthesis are altered in the lumbar spinal cords of SYMP and ES SOD1^G93A^ mice. (**h**) Primary, secondary and conjugated bile acids are significantly increased in the lumbar spinal cords of ES SOD1^G93A^ mice at 10 AM. Statistical comparisons for SOD1^G93A^ mice are compared to wild type (WT) controls (****p = 0.0001, ***p = 0.001, **p = 0.01, and *p = 0.05). Columns not connected by the same letter are significantly (p = 0.001) different from each other. Error bars represent ± SEM.

A compensatory response to cholesterol cacostasis is increased free cholesterol (FC) oxidation to oxysterols and bile acids to enhance its removal^25,26^ (Fig. 1a). To determine if increased FC oxidation occurs during ALS, we first analyzed whole lumbar spinal cords collected from WT and SOD1^G93A^ mice at different stages of disease (N = 8–14/group) for mRNA levels of enzymes that regulate oxysterol synthesis^25^. CYP7A1 mRNA was not detected and CYP27A1 mRNA levels were not altered in SOD1^G93A^ mice. However, significant progressive increases in CYP46A1 and CH25H mRNA levels were observed in SOD1^G93A^ mice vs. WT controls. Notably, CH25H levels were elevated more than 30-fold in end stage (ES) SOD1^G93A^ mice (Fig. 1b). Our results corroborate an earlier finding showing a similar increase in CH25H mRNA in microglia isolated from SOD1^G93A^ mice^27^. Next, we used LC–MS to measure oxysterol levels in the whole lumbar spinal cords of ES SOD1^G93A^ and WT mice (N = 5/group). We found that 25-hydroxycholesterol (25-OHC) was significantly elevated nearly tenfold in the spinal cords of ES SOD1^G93A^ mice (Fig. 1c). Levels of 24S-hydroxycholesterol (24S-OHC) and 27-hydroxycholesterol (27-OHC) were not altered in the spinal cords of SOD1^G93A^ mice (Fig. 1c). Interestingly, several oxysterols derived through the auto-oxidation of cholesterol (Fig. 1d) by reactive oxygen (e.g., OH^**.**^) or nitrogen (ONOO^-^; peroxynitrite)^25^ were also significantly elevated in the spinal cords (Fig. 1e,f) of SOD1^G93A^ mice compared to WT controls. These oxysterols include 7α-hydroxycholesterol (7α-OHC; generated by auto-oxidation or by CYP7A1), 7β-hydroxycholesterol (7β-OHC), 7-ketocholesterol (7-KC), 5α6α-epoxycholesterol (5a6a-EC), 5ß6ß-epoxycholesterol (5b6b-EC) and 5α,6ß-dihydroxycholestanol (THC).

Emerging evidence suggests that BAs synthesis may also occur in the CNS^24,28^; however, BA detection in CNS tissue remains to be demonstrated. We found that the expression levels of CYP7B1 and HSD3B7, enzymes that regulate acidic BA synthesis were significantly upregulated in the spinal cords of ES SOD1^G93A^ mice vs. WT controls (Fig. 1g). In contrast, mRNA levels of CYP8B1, a mediator of neutral BA synthesis was significantly downregulated in symptomatic (SYMP) and ES SOD1^G93A^ mice (Fig. 1g). These findings suggest that oxysterols are metabolized to BAs within the spinal cord of SOD1^G93A^ mice. In the liver, BA synthesis is under circadian regulation^29^; therefore, in order to increase the likelihood of detecting disease-related changes in spinal cord BA levels, we measured primary, secondary and conjugated BAs at two different time points, 10 am and 6 pm, using spinal cords collected from ES SOD1^G93A^ and WT mice (N = 7/group/time point). Significant elevations (up to tenfold) in cholic acid (CA), chenodeoxycholic acid (CDCA), ursodeoxycholic acid (UDCA), β-Muricholic acid (β-MCA), taurocholic acid (TCA), taurochenodeoxycholic acid (TCDCA), taurodeoxycholic acid (TDCA), tauroursodeoxycholic acid TUDCA, Tauro-β-muricholic acid (TBMC), glycocholic acid (GCA), and glycodeoxycholic acid (GDCA) were found in the spinal cords of ES SOD1^G93A^ vs. their WT counterparts at the 10 am (Fig. 1h), but not the 6 pm time point (Supplementary Fig. S1). Collectively, our findings indicate that increased FC oxidation is the spinal cord is indeed a manifestation of disease in ALS. We also demonstrate for the first time that BAs are detected in CNS tissue, and that their levels are likely circadian rhythm dependent.

### Oxidized cholesterol metabolites are potential biomarkers of disease

**Figure 2:**
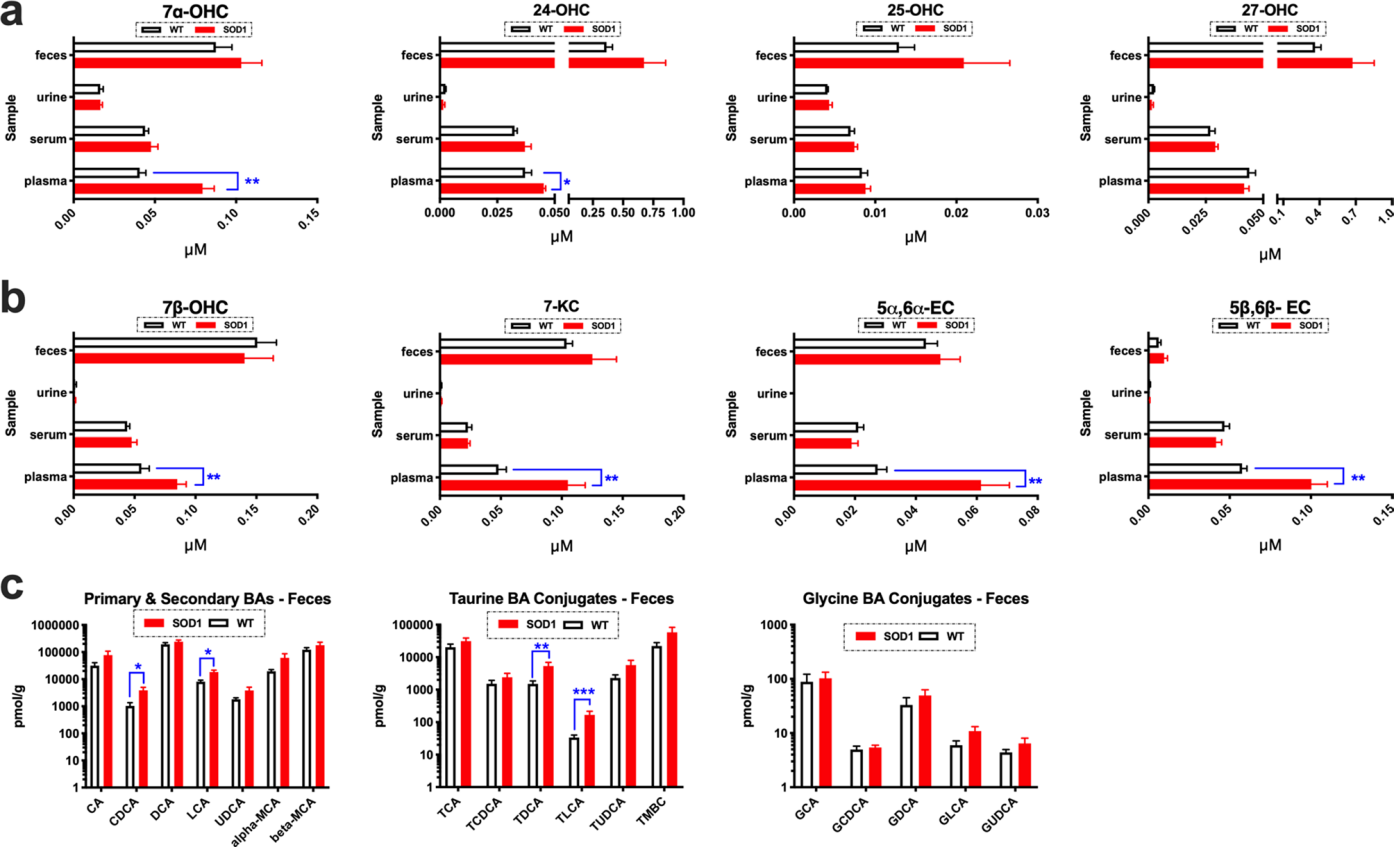
Biomarkers of cholesterol oxidation are detected in the feces, serum and plasma of SOD1^G93A^ mice. (**a**) 7α-OHC and 24-OHC are significantly increased in the plasma of ES SOD1^G93A^ mice. (**b**) Oxysterols generated from the auto-oxidation of sterols including 7β-OHC, 7-KC, 5α6α-EC and 5β6β-EC are significantly elevated in the plasma of ES SOD1^G93A^ mice (note: 7α-OHC is also generated through ROS/RNS exposure). (**c**) Primary (CDCA, LCA) and Taurine bile acid (BA) conjugates (TDCA and TLCA) are significantly increased in the feces of ES SOD1^G93A^ mice. For all comparisons SOD1^G93A^ mice are compared to wild type (WT) controls (****p = 0.0001, ***p = 0.001, **p = 0.01, and *p = 0.05). Error bars represent ± SEM.

Next we measured oxidized cholesterol metabolites in the feces, urine, serum and plasma of ES SOD1^G93A^ mice and WT controls (n = 5/group/sample matrix) to determine if disease changes in cholesterol oxidation found in the spinal cord are also present systemically as potential biomarkers of disease. Oxysterol levels were unaltered in the feces, urine, and serum of ES SOD1^G93A^ mice compared to WT controls. In plasma, however, enzymatically derived oxysterols and auto-oxidized metabolites of cholesterol were significantly elevated in ES SOD1^G93A^ mice compared to WT controls. Specifically, increases in 7αOHC, 24-OHC, 7β-OHC, 7-KC, 5α6α-EC and 5β6β-EC were detected (Fig. 2a, b). Increased fecal BA excretion plays a significant role in removing excess sterols from the body. Consistent with our observations of increased sterols in the spinal cord and plasma of SOD1^G93A^ mice, we also found that BA levels were also significantly elevated in feces during ALS. Specifically, levels of CDCA, LCA, TDCA and TLCA were significantly elevated in the feces of SOD1^G93A^ mice compared to their WT counterparts (Fig. 2c). Collectively, our findings suggest that measuring oxidized cholesterol metabolites in plasma and feces may potentially serve as potential biomarkers of ongoing cholesterol cacostasis that occurs in the spinal cord during disease course in ALS.

### Human and mice show species differences in spinal cord oxidized sterol composition

**Figure 3:**
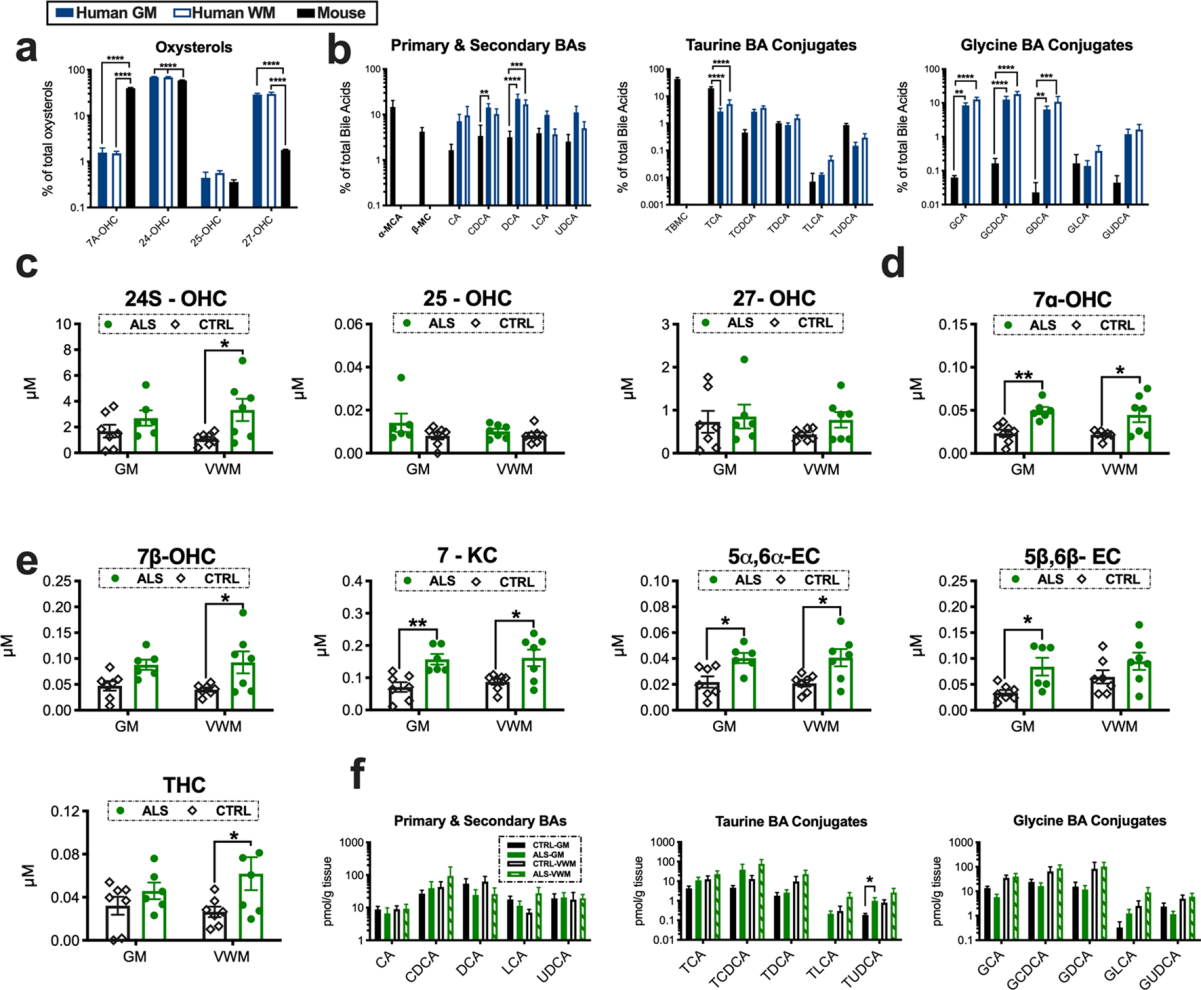
Oxidized metabolites of cholesterol that are elevated in the spinal cords of ALS patients. Humans and mice show comparative differences in spinal cord (GM = grey matter, VWM = ventral white matter) (**a**) oxysterol and (**b**) bile acid composition. (**c**) 24-OHC and (**d**) 7α-OHC levels are increased in the cervical spinal cord homogenates of ALS patients. (**e**) Oxysterols generated from the auto-oxidation of sterols including 7β-OHC, 7-KC, 5α6α-EC, 5β6β-EC and THC are significantly elevated spinal cords of ALS patients. (**f**) Primary, secondary and conjugated bile acid levels in the spinal cords of ALS patients. Statistical comparisons for ALS patient data are relative to age-matched control (CTRL) tissue subtype (****p = 0.0001, ***p = 0.001, **p = 0.01, and *p = 0.05). Columns not connected by the same letter are significantly (p = 0.001) different from each other. Error bars represent ± SEM.

It is unknown if humans and mice have comparative differences in spinal cord cholesterol oxidation. Thus, we compared the relative percentages of enzymatically derived oxysterols and BAs in human control spinal cord grey matter (GM), (ventral white matter) VWM and WT mouse spinal cord tissue homogenates (N = 6–7/group). For enzymatically derived oxysterols significant species differences were observed for 7α-OHC, 24-OHC and 27-OHC (Fig. 3a). Interestingly, levels of 27-OHC were several-fold higher in humans compared to mice. As expected, based on what has been reported for the liver, only α-muricholic acid (α-MCA) and β-MCA were detected in the spinal cords of mice. Species differences in the primary and secondary BAs CDCA and DCA were also found (Fig. 3b). Significant comparative differences in conjugated BAs were also found. Mice showed higher levels of taurine BA conjugates (i.e., TCA), whereas glycine BA conjugates (i.e., GCA, GCDCA, and GDCA) were relatively enriched in humans (Fig. 3b). Collectively, our findings suggest that human and mice oxidize cholesterol differently in the spinal cord.

### Oxidized cholesterol accumulation occurs in the spinal cord of ALS patients

To determine if increased cholesterol oxidation also occurs in ALS patients, we analyzed GM and VWM spinal cord tissue homogenates for levels of enzymatically derived oxysterols, auto-oxidized sterols and BAs (N = 6–7/tissue type/group). In contrast to SOD1^G93A^ mice, 25-OHC levels were not altered, but 24S-OHC levels were significantly increased in the VWM of ALS patients vs. controls (Fig. 3c). Levels of 7α-OHC, an oxysterol generated by both CYP7A and auto-oxidation, was significantly increased in the GM and VWM of ALS patients compared to controls (Fig. 3d). Similar to SOD1^G93A^ mice several auto-oxidized generated sterols including 7β-OHC, 7-KC, 5α6α-EC, 5β6β-EC and THC were significantly increased in the spinal cords of ALS patients compared to controls (Fig. 3e). Not surprisingly, since postmortem tissue samples are collected at variable time points, and our findings in mice suggest BA synthesis in the spinal cord has a circadian rhythm, we did not see significant changes in primary or secondary BA accumulation in the spinal cords of ALS patients. However, a significant elevation in the conjugated BA TUDCA was found in GM of sALS spinal cord tissue samples (Fig. 3f). Importantly, we were able to detect all three classes of BAs within the spinal cord in both mice and humans directly validating for the first time that BAs are present in human CNS tissue. Collectively, our results show that increased cholesterol oxidation is a neuropathological feature of ALS.

### Auto-oxidized metabolites of cholesterol are toxic to human

**Figure 4:**
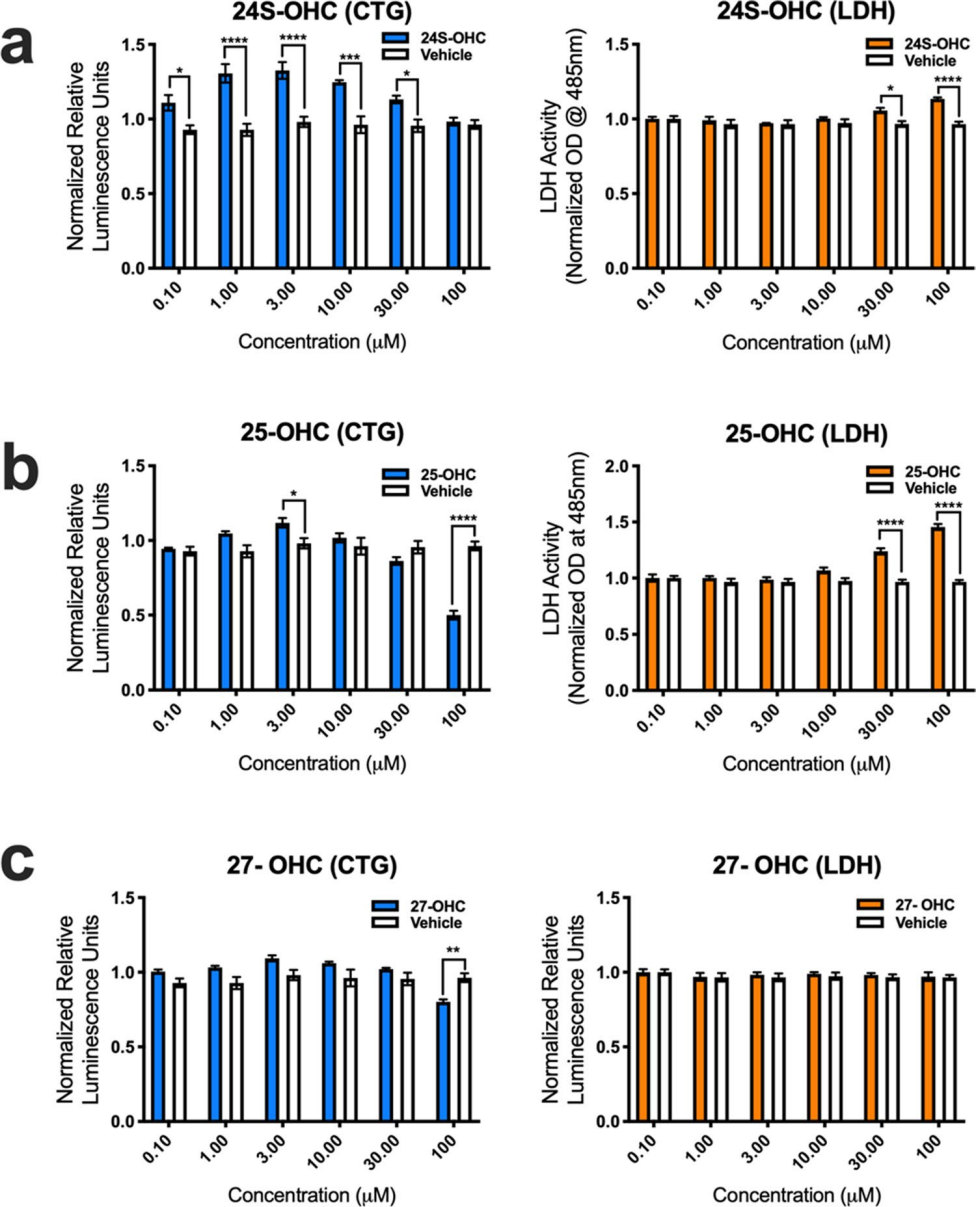
Enzymatically derived oxysterols modulate iPSC derived human MN survival. (**a**) 24S-OHC improves MN viability at low concentrations, but diminishes it at high concentrations as assessed by the cell titer glow (CTG) and lactate dehydrogenase (LDH) assays. (**b**) 25-OHC reduces MN survival at 30 and 100 mM as assessed by the LDH assay. (**c**) 27-OHC adversely affects MN viability at 100 mM as assessed by the CTG assay. All comparisons made to vehicle controls (****p = 0.0001, ***p = 0.001, **p = 0.01, and *p = 0.05). Error bars represent ± SEM.

**Figure 5:**
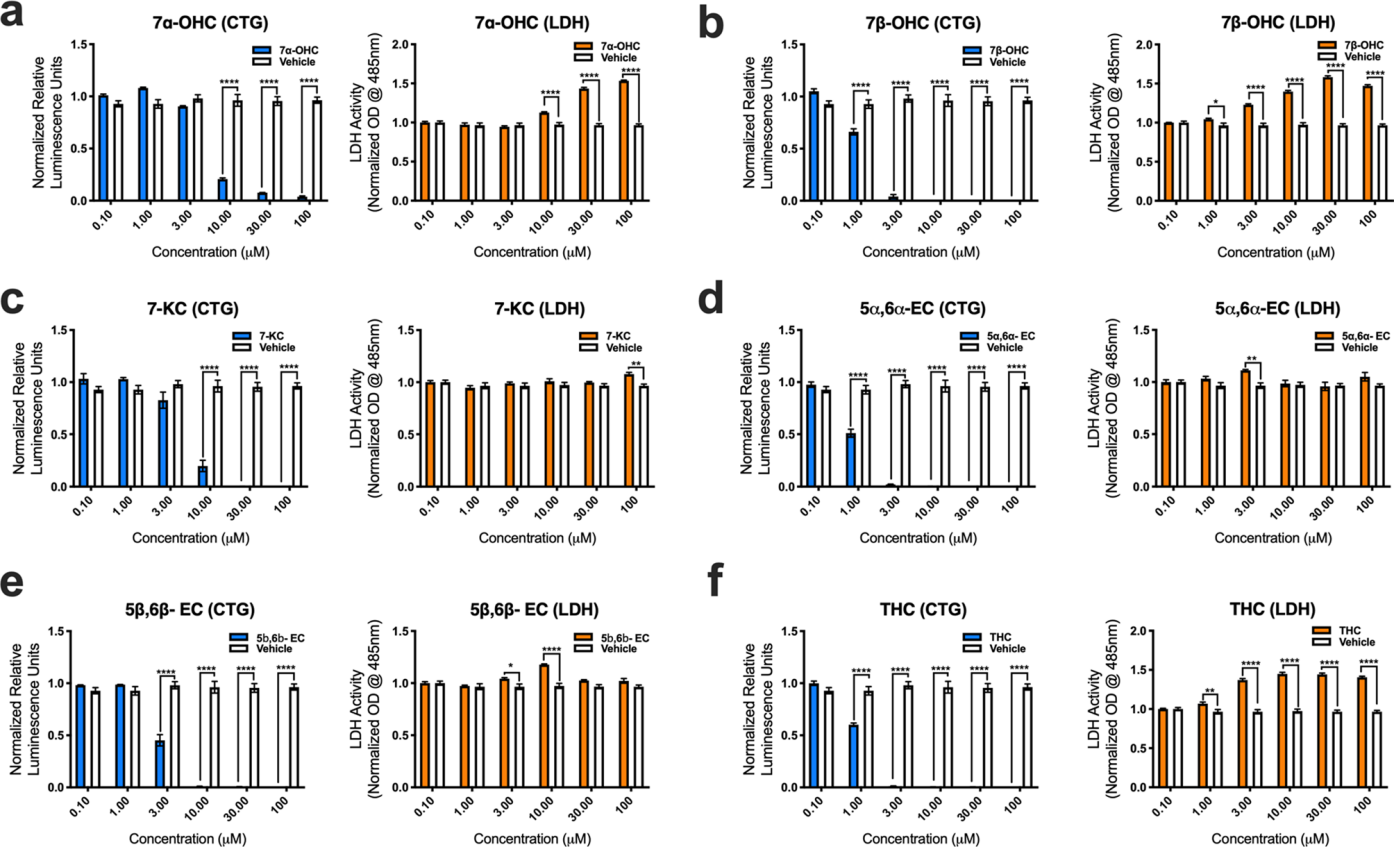
Auto-oxidized sterols adversely affect the viability of iPSC derived human MNs. (**a**) 7α-OHC, (**b**) 7β-OHC, (**c**) 7-KC, (**d**) 5α6α-EC, (**e**) 5β6β-EC and (**f**) TH reduce human MN survival as assessed by the cell titer glow (CTG) and lactate dehydrogenase (LDH) assays. All comparisons made to vehicle controls (****p = 0.0001, ***p = 0.001, **p = 0.01, and *p = 0.05). Error bars represent ± SEM.

To determine if oxidized forms of cholesterol potentially play a role in disease pathogenesis, we assessed human MN (hMN) viability in vitro after adding variable amounts (0.1, 1.0, 3.0, 10, 30 and 100 μM) of enzymatically derived oxysterols (24S-OHC, 25-OHC, 27-OHC and 7α-OHC) and BAs (CA, CDCA, TCDCA, TCA, GCA, GCDCA and GDCA), and auto-oxidized sterols (7β-OHC, 7-KC, 5α6α-EC, 5β6β-EC and THC) to the culture media. Results from CTG and LDH viability assays showed that 24-OHC, 25-OHC, and 27-OHC were either relatively benign or even favorable for hMN survival (Fig. 4.). Interestingly, CTG analysis showed that 24S-OHC significantly improved hMN viability at lower concentrations (0.1, 1.0, 3.0, and 10 μM), but at higher concentrations (30 and 100 μM) it significantly disrupted the membrane permeability of hMN (i.e., LDH release) (Fig. 4a). 25-OHC had similar significant effects on LDH release at higher concentrations (30 and 100 μM) only (Fig. 4b). For 27-OHC, CTG analysis showed that hMN viability was only adversely affected at 100 μM (Fig. 4c). Surprisingly, CTG and LDH analysis showed that BAs even at very high concentrations did not decrease hMN survival (Supplementary Fig. S2). In contrast to enzymatically derived oxysterols, auto-oxidized sterols were relatively more detrimental to hMN survival. 7α-OHC promoted significant hMN demise at (10, 30 and 100 μM) (Fig. 5a). Notably, CTG analysis showed that 7β-OHC, 5α6α-EC, and THC adversely affected hMN viability starting at 1 μM (Fig. 5b,c,d). 7-KC and 5β6β-EC were also damaging to hMN survival starting at 3 and 10 μM respectively (Fig. 5e,f). Collectively, our findings indicate that enzymatically derived oxidized metabolites of cholesterol are relatively non- toxic to hMNs, whereas sterols generated through the auto-oxidation of cholesterol are detrimental to hMN viability.

## Discussion

Similar to other neurometabolic diseases that feature neurodegeneration of the motor system^30^, the unique structural composition of the spinal cord may play a role in determining its relative vulnerability to pathogenic triggers of amyotrophic lateral sclerosis (ALS). Notably, the spinal cord is particularly enriched in cholesterol, and it features the fastest rate of cholesterol synthesis in the CNS—up to fivefold faster than any other region^31^. Indeed, growing evidence suggests that disrupted cholesterol homeostasis (i.e., cholesterol cacostasis) contributes to disease pathogenesis in ALS. Cholesterol cacostasis occurs in the spinal cord during aging^32^, a risk factor for developing ALS, and a number of genes associated with familial ALS are also known to affect cholesterol homeostasis. For example, VCP, FUS and HNRNPA1 regulate levels of HMG-CoA reductase (HMGCR)^8,9,12^, the rate limiting enzyme in cholesterol biosynthesis. Moreover, ANG modulates the neutralization of free cholesterol (FC) to cholesterol esters (CEs)^11^, and VAPB and SIGMAR1 affect intra-organelle cholesterol transport^10,33^. And lastly, CEs are markedly increased in the spinal cords of ALS patients and in SOD1^G93A^ rodent models of disease^17–19^, and inducing cholesterol cacostasis in the SC of normal mice leads to ALS-like lipid neuropathology, astrogliosis, motor neuron (MN) death, and clinical features of ALS^18^.

Sustained FC loading in mammalian cells leads to the activation of several pathological pathways implicated in the demise of MNs including ER stress, ROS generation, mitochondrial dysfunction, inflammation, and cell death^21,25,34^. FC-induced cellular toxicity is averted in part through the hydroxylation of FC to oxysterols, which is dependent upon ER (i.e., CYP7A1, CYP46A1 and CH25H) and mitochondrial (i.e., CYP27A1) resident enzymes. The relative solubility of enzymatically derived oxysterols facilitates their exit from cells^25^. Although oxysterols are elevated in the plasma^22^ and CSF^20^ of ALS patients, it is unknown if increased FC oxidation occurs in the spinal cord during disease. Here, we demonstrate that spinal cord oxysterol accumulation is a pathogenic feature of ALS. Levels of 25-OHC were increased over tenfold in the spinal cords of SOD1^G93A^ mice, but not in ALS patients. The latter finding was unexpected because similar to SOD1^G93A^ mice^27^ CH25H expression is increased in the spinal cords of ALS patients^35^. Detecting disease related changes in patients may be challenging because some oxysterols are produced cyclically^29^ and human tissue samples are often collected at variable circadian time points. Accumulation of 24S-OHC was found in the spinal cords of ALS patients, but not in SOD1^G93A^ mice. Interestingly, however, 24-OHC plasma levels were elevated in SOD1^G93A^ mice, which may be reflective of an earlier change in spinal cord 24-OHC levels. In both ALS patients and SOD1^G93A^ mice 7α-OHC spinal cord levels were elevated. For 27-OHC marked species differences were found with humans being relatively more dependent on this pathway of sterol oxidation. Consistent with these findings mutations in CYP27A1 are associated with cerebrotendinous xanthomatosis, a rare disease which sometimes presents as a clinical mimic of ALS due to upper motor neuron loss in humans^24,36^, but not in mice^37^.

Oxysterols also act as potent signaling molecules to regulate cholesterol synthesis, metabolism, transport, and efflux through their modulation of the transcription factors sterol regulatory element binding protein 2 (SREBP2) and liver X receptors (LXRs). SREBP2 increases cellular cholesterol levels, whereas the activation of LXRs by oxysterols initiates the opposite effect^21,25,31^. Our finding of a marked increase in 25-OHC levels may explain why SREBP2 activation progressively decreases with disease in SOD1^G93A^ mice^18^, as 25-OHC precludes the proteolytic processing of SREBP2 into its activate form^38^. Moreover, elevated 24S-OHC levels may account for reductions in HMGRC that are observed in some ALS patients^18^; 24S-OHC directly triggers the proteolytic degradation of HMGCR^21^. Sustained oxysterol activation of LXRs also promotes the expression of members of the ATP-binding cassette (ABC) transporter super-family, which expel cholesterol from cells. Thus, increased oxysterol signaling may also potentially explain reported increases in ABCG1 and ABCA1 found in SOD1^G93A^ mice^18,39^. Notably, disruption of LXR signaling in mice leads to an ALS phenotype^40^ and gene variants in LXR are associated with altered disease progression in ALS patients^5^.

Oxysterols undergo further hydroxylation and oxidation to generate bile acids (BAs), a metabolic route that is under circadian control and reportedly circumscribed to the liver^41^. Several lines of evidence, however, suggest that BAs are synthesized locally within the CNS and that they have important roles in modulating MN viability. For example, cholestenoic acids activate LXRs to modulate MN survival in vitro and mutations in enzymes that regulate the synthesis of BA intermediates are associated with neuromuscular diseases including hereditary spastic paresis type 5, cerebrotendinous xanthomatosis, and adult-onset motor neuropathy^24^. Indeed, our current findings suggest that BA synthesis does occur in the spinal cord, and that it is also upregulated during disease course in ALS. Interestingly, in SOD1^G93A^ mice expression of CYP7B1, a regulator of acidic BA synthesis was increased in the spinal cord. This may be part of a compensatory response to avert disease because it is thought that reduced levels of acid BA precursors in the CSF of ALS patients contributes to disease progression^20^. Our measurement of BA levels showed that several primary, secondary and conjugated BAs were increased in the spinal cords of SOD1^G93A^ mice in a circadian dependent manner. This observation highlights the importance of taking circadian rhythms into consideration when trying to understand pathogenic mechanisms of disease. Not surprisingly, since human tissue collected at autopsy often occurs at varying times, BA levels in the spinal cords of ALS patients and controls were highly variable. Non-enzymatic or unregulated oxidation also occurs when sterols are inadvertently exposed to reactive oxygen or nitrogen species (e.g., OH^**.**^ and ONOO^-^) or to immune cell oxidizers such as H_2_0_2_ and HOCl^25^. This potential route of sterol oxidation seems likely during ALS because ONOO^-^ accumulation^42^ and neuroinflammation are robust features of disease^43^. Indeed, we found that several auto-oxidized sterols were elevated in the spinal cords of ALS patients and SOD1^G93A^ mice. Moreover, we also found that several auto-oxidized sterols, as well as enzymatically derived oxysterols and BAs were also elevated in the plasma and feces of SOD1^G93A^ mice. Thus, oxidized sterols may potentially serve as biomarkers of ongoing cholesterol cacostasis during ALS.

It is unclear if increased sterol oxidation is a compensatory response to disease (to reduce FC levels) or if it contributes to disease pathogenesis. Several studies suggest that oxidized sterols may adversely affect the viability of human MNs. For example, 24S-OHC triggers necroptosis in mouse neurons^44^, a cell death pathway implicated in ALS^45^. In addition, in rat organotypic brain slice cultures 24-OHC and 25-OHC markedly reduced ChAT-positive neuronal survival^46^, and 25-OHC reduces the survival of SOD1^G93A^ NSC34 motor neuron like cells^22^. Furthermore, at high concentrations BAs function as membrane solubilizers, induce mitochondrial dysfunction and cause death in several cell types^34^. Moreover, sterols generated through auto-oxidation induce cell lysosomal and mitochondrial dysfunction^47^, and oxiapoptophagy^48^—a form of cell death involving simultaneous oxidative stress, apoptosis, and autophagy. Interestingly, in our experiments enzymatically derived oxysterols and BAs were relatively harmless to human MNs; and in some instances (e.g. 24-OHC) even improved human MN viability. In contrast to the enzymatically derived oxysterols; however, we found that several auto-oxidized generated sterols did adversely affected human MN survival. Collectively, these findings suggest that enzymatically regulated cholesterol oxidation during ALS may be a compensatory response to avert cholesterol cacostasis; whereas, exposure to auto-oxidized derivatives of cholesterol likely contributes to MN death. These findings should be interpreted with caution; however, because oxidized sterols in our experiments were not delivered to MNs using intracellular carrier proteins (e.g., oxysterol or fatty acid binding proteins) which would enable sterols to more efficiently affect MN physiology. Moreover, the indirect effect that these oxidized sterols have on MN survival through their effects on additional cell types that contribute to disease pathogenesis remains largely unknown. Nevertheless, our findings suggest that therapeutic approaches that restore CNS cholesterol metabolism to its optimal homeostatic state (i.e., eustasis) may lead to a disease-modifying treatment for ALS.

## Materials and methods

### Human tissue samples

Cervical spinal cord segments from six male patients who died of respiratory failure caused by advanced ALS were used in our analyses. The control cervical spinal cord segments used in our experiments came from seven age-matched male individuals without evidence of neurological disease. All human tissue samples were obtained from the NICHD Brain and Tissue Bank for Developmental Disorders at the University of Maryland, Baltimore, MD, contact HHSN275200900011C, Ref. No. N01-HD-9-0011.

### Animals

Transgenic male ALS mice that express the mutant SOD1^G93A^ transgene at high levels (B6SJL background strain) were divided equally among groups. Mutant SOD1^G93A^ gene copy number and protein expression were confirmed by PCR and western blot analysis, respectively. Animals were housed under light:dark (12:12 h) cycles and provided with food and water ad libitum. At 75 days of age, food pellets were place on the cage floor to facilitate access and consumption. Mice were scored daily into the following phases: symptomatic (SYMP; median age of 82 days) = abnormal hind limb splay, end stage (ES; median age of 103 days) = onset of limb paralysis (typically hind limb), and moribund (MB; median age of 122 days) = unable to right themselves within 30 s after being placed on their backs. Wild type mice used were unaffected siblings from the same breed pairs. All samples taken from mice were collected between 9:45 and 10:15 AM unless stated otherwise to avoid variation due to circadian fluctuations in metabolism. This time point was selected because previously we showed that increased corticosterone signaling and glycogen accumulation—metabolic consequences of increased BA signaling—occur in SOD1^G93A^ mice during this sampling period^49,50^. At sacrifice all mice were perfused with cold 1X PBS (pH 7.4); the lumbar spinal cords were removed, flash frozen in liquid nitrogen and then stored at -80C. The N values and tissue samples used from each mouse is described in detail below for each assay. All experimental procedures and methods were performed in accordance with protocols and regulations approved by Sanofi Genzyme’s Institutional Animal Care and Use Committee (IACUC)***.***

### Experimental design and statistics

All experiments were carried out in compliance with the ARRIVE guidelines. The experimental design for each assay is described in its subsection below. Normality was determined using the Shapiro–Wilk normality test. Data sets that failed the normality test were analyzed with a Mann–Whitney test. A two-tailed unpaired t-test was used to compare data sets that passed the normality test and that had equal variances. If data set variances were significantly different, then an unpaired t-test with Welch’s correction was used to compare groups that passed the normality test. Statistical tests comparing multiple groups were performed using a 1-way analysis of variance (ANOVA) followed by a Dunnett’s multiple comparison post hoc test to find differences between group means. A value of p < 0.05 was considered statistically significant. All statistical tests were performed using GraphPad Prism Software 8.0.

### Sterol analysis of mouse and human samples

Oxysterols analysis was performed by Biocrates Life Sciences AG. Tissue homogenates for oxysterol analysis were prepared by dounce homogenizing (20 strokes) samples: human cervical spinal cord tissue grey and white matter tissue (n = 6–7/tissue type/group), mouse lumbar spinal cord tissue (n = 5/group), mouse feces (25 mg; n = 5/group) in a buffer (20 × vol/weight) composed of 10 mM Hepes, 10 mM NaCl, 1 mM KH_2_PO_4_, 5 mM NaHCO_3_, 5 mM EDTA, 1 mM CaCl_2_ and 0.5 mM MgCl_2_. Homogenates were placed on ice for 10 min and then centrifuged at 6300 X g at 4 °C for 10 min. Oxysterols were extracted from the tissue homogenate supernatants, mouse plasma (100ul; n = 5/group) and mouse serum (100 ul; n = 5/group) with methanol using the BIOCRATES Kit filter plate, which was preloaded with an internal standard mixture. The extract was then subjected to alkaline hydrolysis to release oxysterols from their respective esters. After neutralization the metabolites were determined by UHPLC-tandem mass spectrometry (LC–MS/MS) with Multiple Reaction Monitoring (MRM) in positive mode using a SCIEX API QTRAP5500 mass spectrometer with electrospray ionization.

Bile acid (BA) analysis was carried out by Metabolon, Inc on spinal cord tissue samples (175–200 mg/sample). For human samples, grey and white matter tissue segments (n = 6–7/tissue type/group) from the cervical spinal cord were analyzed separately. For mouse experiments entire spinal cords were used (n = 14/group/time point). Spinal cord tissue from 2 different mice (80–100 mg tissue per mouse) were pooled together to generate 1 sample; thus, reported BA levels were generated from n = 7/group/time point. Deuterium-labeled internal standards were added to tissue samples and the mixture was solubilized in methanol followed by a crash extraction. Samples were dried under nitrogen and reconstituted in methanol:deionized water and then transferred to silanized autosampler inserts for LC/MS/MS analysis and then injected onto an Agilent Stable Bond C18 reverse phase column connected to an Applied Biosystems 4000 QTRAP. The analytes were ionized via negative electrospray and the mass spectrometer was operated in the tandem MS mode. The absolute concentration of each bile acid was determined by comparing the peak to that of the relevant internal standard. Analysis included the quantification of primary BAs (cholic acid, CA; chenodeoxycholic acid, CDCA), secondary BAs (deoxycholic acid, DCA; lithocholic acid, LCA; ursodeoxycholic acid, UDCA; α-muricholic acid, α-MCA; β-Muricholic acid, β-MCA), taurine BA conjugates (taurocholic acid, TCA; taurochenodeoxycholic acid, TCDCA; taurodeoxycholic acid, TDCA; taurolithocholic acid, TLCA; Tauro-β-muricholic acid, TBMC) and glycine BA conjugates (glycocholic acid, GCA; glycochenodeoxycholic acid, GCDCA; glycodeoxycholic acid, GDCA; glycolithocholic acid, GLCA; and glycoursodeoxycholic acid, GUDCA).

### Quantitative reverse transcription–polymerase chain reaction

Total RNA was extracted with Ambion MagMAX-96 RNA isolation kit (Applied Biosystems) from the lumbar spinal cords of mice (N = 8–14/group), reverse-transcribed, amplified with TaqMan One-Step RT-PCR Master Mix Kit (Applied Biosystems), and analyzed on an ABI PRISM 7500 Real-Time PCR System (Applied Biosystems). Primer probes purchased from ThermoFisher Scientific were used to quantify the expression levels for CYP7A1 (Mm00484150_m1), CYP46A1 (Mm00487306_m1), CH25H (Mm00515486_s1), CYP27A1 (Mm00470430_m1), HSD3B7 (Mm01159156_g1), CYP8B1(Mm00501637_s1), CYP7B1(Mm00484157_m1), and CYP39A1 (Mm00517060_m1), Expression analysis was normalized to PPIA (Mm02342430_g1) mRNA levels.

### Sterol preparation for in vitro studies

*Oxysterols* (24S-OHC, sc-471350; 25-OHC, sc-214091B; 27-OHC, sc-358756; and 7α-OHC, sc-210659), and auto-oxidized sterols (7β-OHC, sc-210655; 7-KC, sc-210630; 5α6α-EC, sc-214687; 5β6β-EC, sc-214688; and THC, sc-474630) were purchased from Santa Cruz Biotechnology and were formulated in stock ethanol solutions at 10 mM. Bile acids were purchased from either Sigma Aldrich (GCA, G2878-1G; TCA, T4009-1G; TCDCA, T6260-1G), Cayman Chemical (GCDCA, 16564-43-5) or Santa Cruz Biotechnology (CDCA, sc-280637; TCDCA, sc-281162; GDCA, sc-280755) and were formulated in PBS stock solutions at 20 mM.

### Human motor neuron viability studies

Experiments were carried out by BrainXell, Inc. using human iPSC-derived motor neurons from a normal control patient (WC-30 line). The culture medium was composed of a 1:1 mixture of Neurobasal Medium (Life Technologies #21103-049) and DMEM/F12 Medium (Life Technologies #11330-032) supplemented with the following: 1X B27 Supplement (Life Technologies #17504-044), 1X N2 Supplement (Life Technologies #17502-048), 0.5 mM GlutaMAX (Life Technologies #35050-061), 15 μg/mL Geltrex (Life Technologies #A1413201), 200 μM ascorbic acid, 10 ng/mL BDNF (Peprotech #450-02), and 1X BrainXell Motor Neuron Supplement. Neurons were seeded (Day 0) at 100 μL/well onto white-walled, clear-bottom, PDL-coated 96-well plates (Greiner Bio-One #655944) at a concentration of 10,000 neurons/well. After seeding, plates were placed in a humidified chamber inside a standard cell culture incubator with 5% CO2 at 37 °C. On Day 7, 10 µL of the corresponding treatment solution was added to each well. For the negative control condition (“vehicle”), medium alone was added. An additional 90 µL of fresh culture medium was then added to all wells to reach a final volume of 200 µL/well. Each treatment was tested in triplicate in an 8-point dose-dependent manner. On Day 10, the plates in each respective group were processed to determine ATP concentration using CellTiter-Glo 2.0 assay (Promega, G9242) according to the manufacture’s protocol. All plates and reagents were brought to room temperature before beginning the assay. First, 100 μL of medium was gently removed from each well. Next, 100 μL of the CellTiter-Glo buffer/luciferase solution was added to the wells. After 2 min of shaking at 300 rpm and 10 min of stabilization, the relative luminescence signal was measured using a plate reader (Tecan). Plates were processed in a staggered manner to eliminate any differences in assay timing. The release of lactate dehydrogenase (LDH) into the culture medium was also measured on day 10. LDH is normally present only in small amounts in the culture medium but increases in concentration when released from dead or dying cells. The LDH concentration was measured according to the manufacture’s protocol (Lactate Dehydrogenase Activity Assay Kit, Sigma #MAK066-1KT). Briefly, 50 μL of culture medium (supernatant) was gently removed from each well and then transferred to a new clear-bottom plate. Assay reagents were then added to reach a final volume of 100 μL. The plates were incubated at 37 °C in an incubator for 30 min. The absorbance signal at 485 nm, which was proportional to the LDH concentration, was then measured using a plate reader (Tecan).

## Supplementary Information

Supplementary Fig. S1.

Supplementary Fig. S2.

Supplementary information.

## Data Availability

The datasets generated during and/or analyzed during the current study are available from the corresponding author on reasonable request.
