# Supplementary figures and images for: Sterol auto-oxidation adversely affects human motor neuron viability and is a neuropathological feature of amyotrophic lateral sclerosis
Source: Sci Rep. 2021 Jan 12;11:803. doi: 10.1038/s41598-020-80378-y (PMC7804278; doi:10.1038/s41598-020-80378-y)

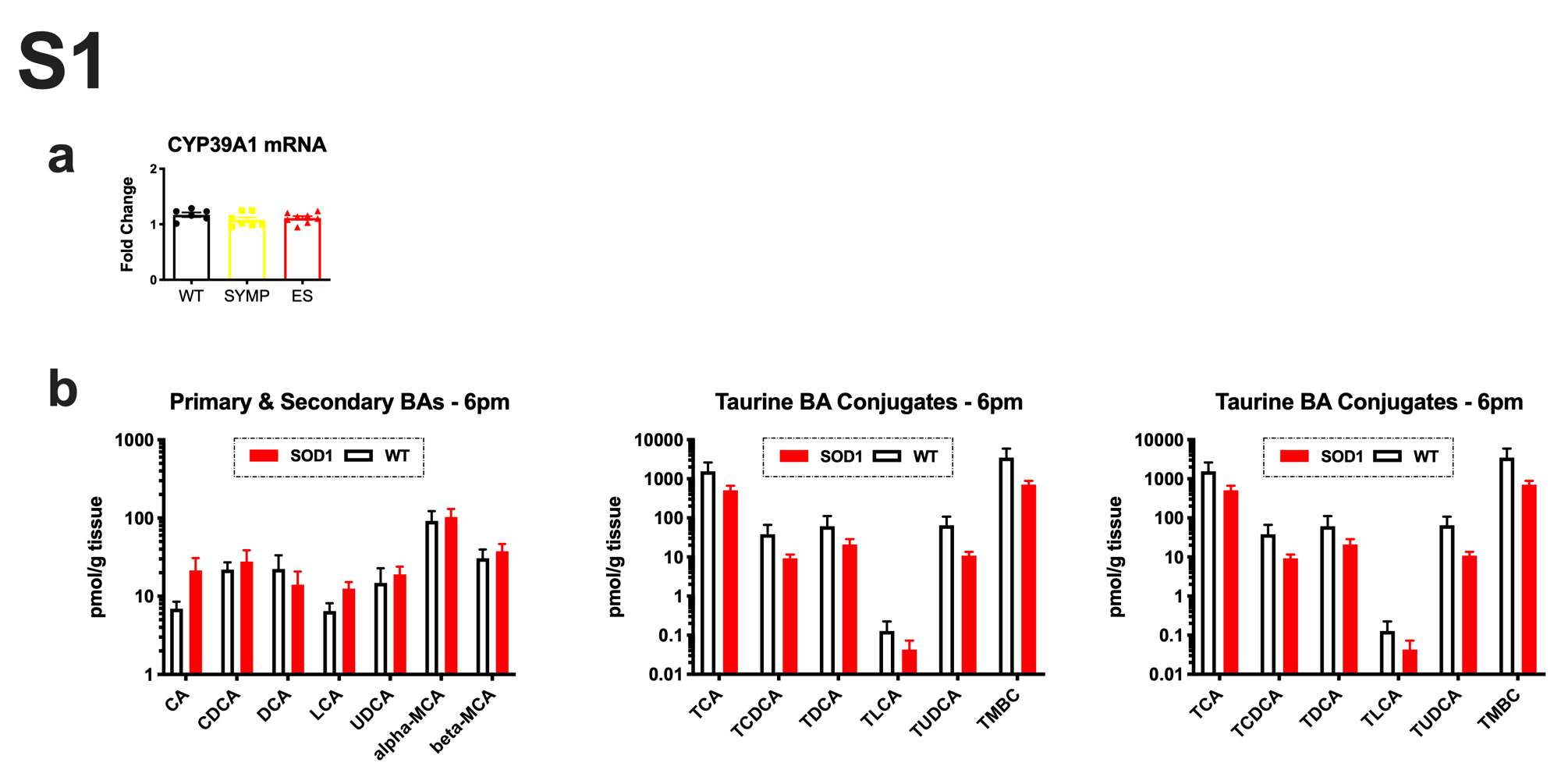

Supplement: Supplementary file 1 — Supplementary Fig. S1. [file 41598_2020_80378_MOESM1_ESM.tiff]

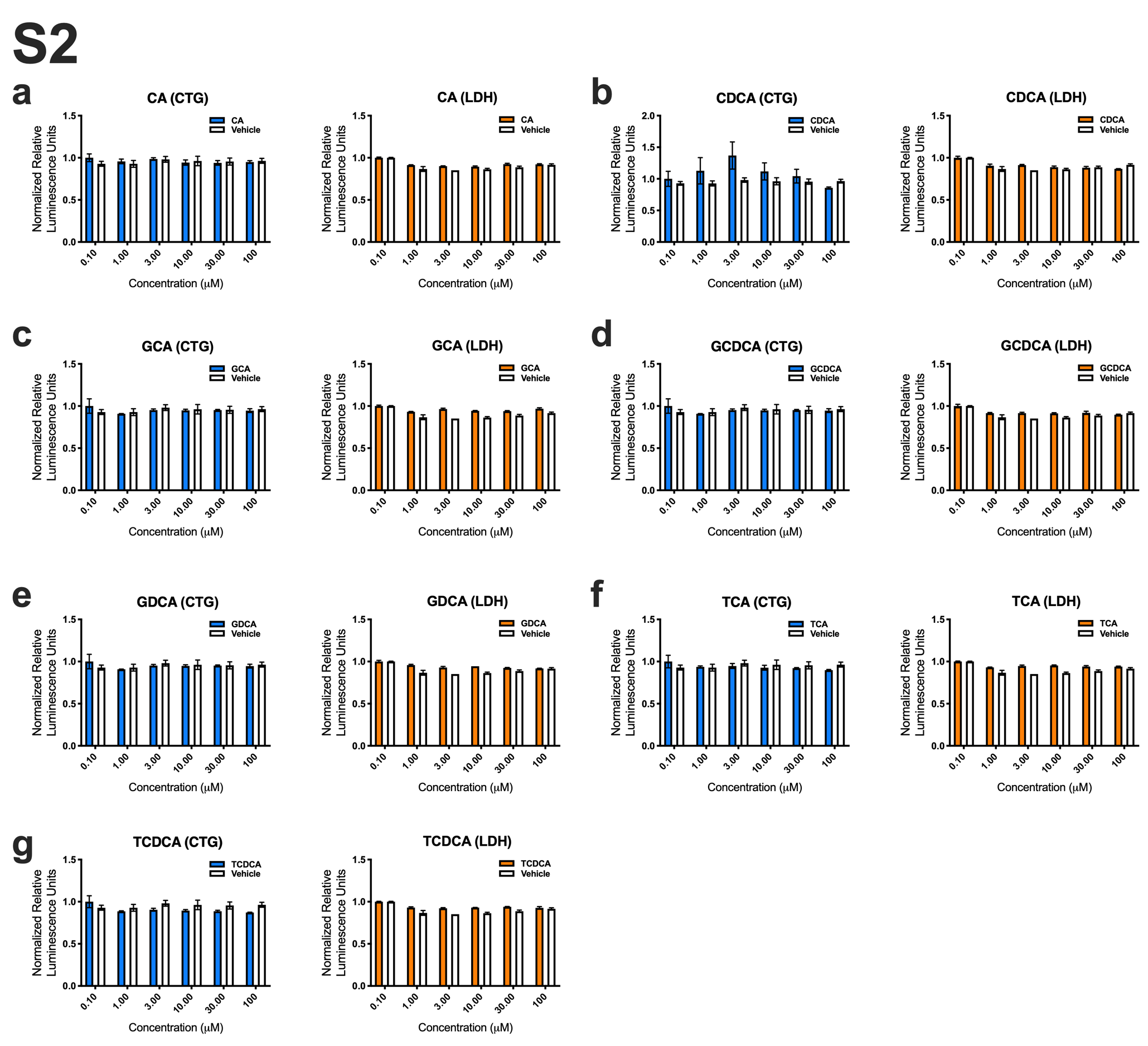

Supplement: Supplementary file 2 — Supplementary Fig. S2. [file 41598_2020_80378_MOESM2_ESM.tiff]
