# Supplementary material for: Sterol auto-oxidation adversely affects human motor neuron viability and is a neuropathological feature of amyotrophic lateral sclerosis
Source: Sci Rep. 2021 Jan 12;11:803. doi: 10.1038/s41598-020-80378-y (PMC7804278; doi:10.1038/s41598-020-80378-y)
Supplement: Supplementary file 3 — Supplementary information. [file 41598_2020_80378_MOESM3_ESM.docx]

**Supplementary Information**

**Sterol auto-oxidation adversely affects human motor neuron viability and is a neuropathological feature of amyotrophic lateral sclerosis**

James C. Dodge^1^, Jinlong Yu^1^, S. Pablo Sardi^1^ and Lamya S. Shihabuddin^1^

**^1^Rare and Neurological Diseases Therapeutic Area, Sanofi R+D, Framingham MA 01701, USA**

**Figure Legends**

**Figure S1. Bile acids in the spinal cords of SOD1^G93A^ mice at 6 pm. (a)** CYP39A1 levels in the lumbar spinal cord of symptomatic (SYMP) and end stage (ES) SOD1^G93A^ mice. **(B)** Primary, secondary and conjugated bile acids are not altered in the lumbar spinal cords of ES SOD1^G93A^ mice at 6 PM. Statistical comparisons for SOD1^G93A^ mice are compared to wild type (WT) controls (****p = 0.0001, ***p = 0.001, **p = 0.01, and *p = 0.05). Columns not connected by the same letter are significantly (p = 0.001) different from each other. Error bars represent ± SEM.

**Figure S2.** **Bile acids do not adversely affect iPSC derived human MN survival.** **(a)** CA, **(b)** CDCA, **(c)** GCA, **(d)** GCDCA, **(e**) GDCA, **(f)** TCA and **(g)** TCDCA do not negatively affect human MN survival as assessed by the CTG and LDH assays. All comparisons made to vehicle controls (****p = 0.0001, ***p = 0.001, **p = 0.01, and *p = 0.05). Error bars represent ± SEM.
